# Association between socioeconomic status and longitudinal sleep quality patterns mediated by depressive symptoms

**DOI:** 10.1093/sleep/zsab044

**Published:** 2021-02-25

**Authors:** Ga Bin Lee, Hyeon Chang Kim, Ye Jin Jeon, Sun Jae Jung

**Affiliations:** 1 Department of Public Health, Yonsei University Graduate School, Seoul,South Korea; 2 Cardiovascular and Metabolic Diseases Etiology Research Center, Yonsei University College of Medicine, Seoul, South Korea; 3 Department of Preventive Medicine, Yonsei University College of Medicine, Seoul,South Korea; 4 Department of Epidemiology, Harvard T. H. Chan School of Public Health, Boston, MA,USA

**Keywords:** socioeconomic status, sleep quality, trajectory analysis, Pittsburgh Sleep Quality Index

## Abstract

**Study Objectives:**

We aimed to examine whether associations between socioeconomic status (SES) and longitudinal sleep quality patterns are mediated by depressive symptoms.

**Methods:**

We utilized data on 3347 participants in the Korean Genome and Epidemiology Study aged 40–69 years at baseline from 2001 to 2002 who were followed up for 16 years. A group-based modeling approach was used to identify sleep quality trajectories using the Pittsburgh Sleep Quality Index (years 2, 6, 8, 10, and 12). Educational attainment (college graduated or less), monthly household income (≥$2500 or less), and occupation (unemployed, manual labor, and professional labor) at baseline (year 0) were used for analyses. Depressive symptoms were assessed using Beck’s Depression Inventory at year 4. Associations between SES and sleep quality patterns were examined using a multinomial logistic regression model. The mediation effect of depressive symptoms was further examined using PROC CAUSALMED.

**Results:**

We identified five distinct sleep quality trajectories: “normal-stable” (*n* = 1697), “moderate-stable” (*n* = 1157), “poor-stable” (*n* = 320), “developing to poor” (*n* = 84), and “severely poor-stable” (*n* = 89). Overall, associations between SES levels and longitudinal sleep patterns were not apparent after full adjustment for sociodemographic and lifestyle factors measured at baseline. Depressive symptoms, however, tended to fully mediate associations between SES levels and sleep quality patterns (odds ratio range for indirect effects of depressive symptoms: for education, 1.05-1.17; for income, 1.05-1.15).

**Conclusion:**

A significant mediating role for depressive symptoms between SES levels and longitudinal sleep quality warrants consideration among mental healthcare professionals.

**Statement of Significance**

We found five discrete longitudinal sleep quality patterns over 12 years: “normal-stable,” “moderate-stable,” “poor-stable,” “developing to poor,” and “severely poor-stable.” Overall, associations between socioeconomic status (SES; i.e. education or household income) and longitudinal sleep quality patterns were not apparent. Depressive symptoms, however, were found to fully mediate associations between SES and longitudinal sleep quality patterns. Our findings may provide evidence regarding the detrimental effects of mental health vulnerability on longitudinal sleep quality in people with lower SES.

## Introduction

Poor sleep quality has been shown to be associated with the prevalence or incidence of cardiovascular diseases [1, 2], mortality [3], and depression later in life [4]. From 2006 to 2013, the prevalence of physician-diagnosed insomnia increased from 3.9% to 6.2% according to a previous study using US Medicare data [5]. Meanwhile, in another study using a multistage random sampling method, 20% of a South Korean sample reported insomnia symptoms (i.e. difficulty in waking up or getting back to sleep at night) [6].

Socioeconomic status (SES) consists of complicated structures that reflect an individual’s access to culturally relevant resources needed to succeed in society [7]. Since SES is similar in nature to latent variables, such as mood or well-being, SES cannot be measured directly; as a result, income, education attainment, and occupation are frequently used to assess SES [7]. Interestingly, SES levels have been shown to be associated with sleep quality [8–10]: one cross-sectional study using data on 160 000 American individuals found that people with lower educational attainment or lower income had more frequent sleep complaints [9]. In another study of 5500 individuals representative of the Finnish population (ages 30-79 years), lower household income or lower education was significantly associated with disturbed sleep during the preceding 30 days [10].

Less educated people are more likely to have fewer economic or social resources. As a result, these people are at a greater risk of experiencing stressful life events, which may lead to depression or anxiety disorder [11]. Meanwhile, insomnia is a common comorbid condition of depressive symptoms [4, 12], and people with insomnia are more likely to exhibit high levels of psychiatric distress or somatic anxiety [13]. However, to our knowledge, few studies have investigated the complex aspects of SES, depressive mood, and sleep quality simultaneously at a large population level. Moreover, the methods used in measuring sleep quality in previous studies were unable to fully examine the changes in sleep quality: several previous studies on sleep quality have used a single appraisal approach at baseline [3, 14] or evaluated linear changes at two time points [15]. Although a previous study investigated changes in insomnia status over time in the general Canadian population [16], it only used a small sample size (*n* = 250) and had a relatively short follow-up period of only 3 years.

Longitudinal sleep quality patterns have rarely been investigated due to a few practical challenges, such as a limited number of sleep quality data and limited observation time [3, 14, 16]. Moreover, sleep quality has not been evaluated in relation to SES and mental health simultaneously. Therefore, we aimed to document distinct sleep quality patterns in middle-aged Korean adults using data measured continually over 12 years. In addition, we sought to examine associations among baseline SES factors and respective longitudinal sleep quality patterns. Moreover, we attempted to determine whether depressive symptoms mediate the associations between SES and longitudinal sleep quality patterns.

## Methods

### Data and study participants

**Figure 1.**
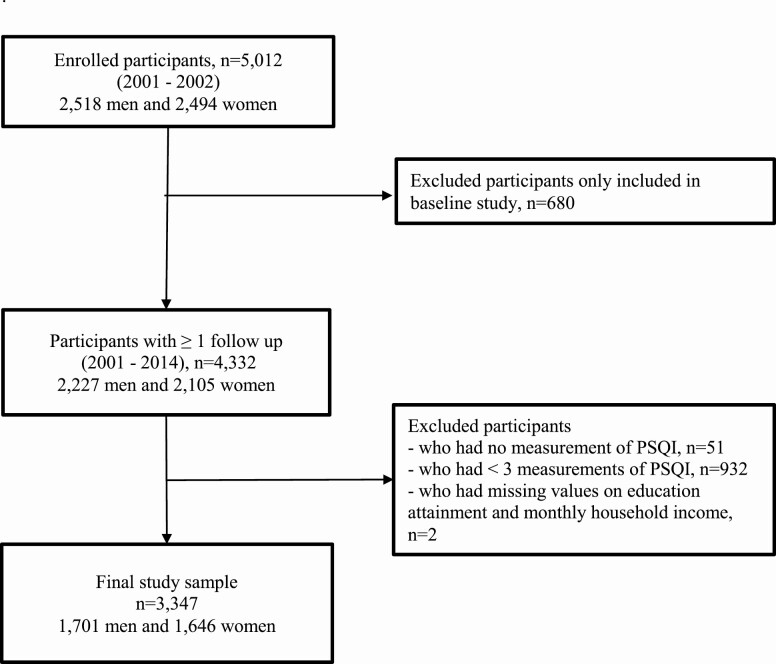
Flow chart of the selection of study participants in the KoGES, Ansan.

The participants of the present study were part of the Korean Genome and Epidemiology Study (KoGES), which is an ongoing, population-based cohort study. Detailed information on the study design and aims of the KoGES has been previously reported [17]. Briefly, the aims of the KoGES are to investigate the genetic and environmental causes of chronic diseases (e.g. hypertension, metabolic syndrome, and type 2 diabetes mellitus) in Korea and to establish a genome epidemiological platform with a health database and biobank [17]. In total, 5012 participants aged 40–69 years with residence in the Ansan region, an industrialized community, were recruited between 2001 and 2003. Registered residents in Ansan were randomly selected and contacted via mail, telephone, or home visits for selecting representative samples [17]. The distributions of age and sex among recruited participants were similar to those who were not recruited. All participants underwent baseline examinations and were followed up biennially up to the seventh follow-up survey. In the present study, we used the participants’ baseline examination data and data from five repeated surveys of sleep quality information, which were conducted at years 2, 6, 8, 10, and 12. The attrition rates were 80.3% (*n* = 4023), 64.9% (*n* = 3255), 65.1% (*n* = 3262), 60.9% (*n* = 3052), and 59.9% (*n* = 3000) at years 2, 6, 8, 10, and 12, respectively. We excluded participants who were only included in the baseline study (*n* = 680), those without sleep quality data (*n* = 51), or those included in less than three surveys on sleep quality (*n* = 932). Lastly, participants who had missing data on educational attainment and monthly household income (*n* = 2) were also excluded. Hence, only 3347 participants (1701 men and 1646 women) were included in the final analyses. Details on the exclusion criteria are shown in Figure 1. The final study samples completed three (*n* = 464, 13.9%), four (*n* = 773, 23.1%), or five (*n* = 2110, 63.0%) of the sleep quality surveys.

### Assessment of exposure variables: SES

Participants were asked to answer questions about their educational attainment and monthly household income. Educational attainment was initially queried as “elementary or less,” “middle school,” “high school,” “college,” “university,” or “graduate school or above.” Monthly household income was initially queried as follows: “<$400,” “$400 to <800,” “$800 to <$1250,” “$1250 to <$1650,” “$1650 to <$2500,” “$2500 to <$3300,” “$3300 to <$5000,” or “≥ $5000,” which were converted from Korean won to US dollar. For feasible interpretation, first, educational attainment was divided into two groups (lower attainment: high school or less; higher attainment: college or above) considering the high enrollment rates (passed 90% since 1994) for high schools in Korea [18]. Second, monthly household income was also divided into two groups (lower income: <$2500; higher income: ≥$2500). We utilized $2500 (3 000 000, South Korean Won [KRW]) as a proxy for the highest tertile of household income, consistent with previous studies [19, 20]. Lastly, occupation was classified into “unemployed,” “manual labor,” and “professional labor” [21]. Homemakers, etc. were classified as “unemployed”; office workers and expertise were classified as “professional laborers”; others were classified as “manual laborers.” (Supplementary Table 1).

### Assessment of outcomes: sleep quality trajectory

The overall sleep quality was assessed using the Pittsburgh Sleep Quality Index (PSQI), which has been validated in the Korean population (Cronbach’s alpha coefficient of 0.84, sensitivity of 0.94, and specificity of 0.84, with a cutoff value of 8.5) [22]. The PSQI is an 18-item measure of self-reported sleep quality and duration that evaluates sleep over the previous 4 weeks. Global PSQI scores (range: 0-21) consist of seven subdomain component scores, including those for sleep quality perception, sleep latency, sleep duration, sleep efficiency, sleep disturbance, sleep medication use, and daytime dysfunction, with the scores of each item ranging from 0 to 3. Higher PSQI scores indicate worse sleep quality and duration. Normally, a PSQI total score <5 is considered the threshold for “good” sleep quality, whereas a PSQI total score ≥5 indicates “poor” sleep quality [23].

### Assessment of potential mediators: depressive symptom

We assumed depressive symptoms at year 4 as a potential proxy mediator between SES and longitudinal sleep quality patterns. Depressive symptoms were assessed using Beck’s Depression Inventory (BDI), which has been validated in the South Korean population [24] (Cronbach’s alpha coefficient of ≥0.87, sensitivity of 0.78, and specificity of 0.77, with a cutoff value of 13). The BDI consists of 21 items, and each item is rated on a 3-point Likert scale, with a total score of 63. This tool is used to assess depressive symptoms over the most recent 2 weeks. Higher scores indicate severe depressive symptoms.

### Assessment of covariates

In the KoGES, all participants provided their demographic information, personal health history, and lifestyle factors. Marital status was categorized into “never married,” “currently married,” and “separated, divorced, or widowed.” The number of family members was reported. More than 15.0 metabolic equivalent (MET) hours/week were categorized as “moderate exercise.” [25]. In terms of drinking and smoking status, participants were classified as current, former, or none. Baseline insomnia symptoms were determined by positive responses to the question, “Do you have insomnia?,” and baseline depressive symptoms were determined by positive responses to the question, “Are you feeling depressed?” Physical examinations, including measurements of body size, composition, and blood pressure, were conducted by trained research personnel. Biochemical analyses of blood samples obtained after an 8-hour fast were performed.

### Statistical analyses

Sleep quality trajectories were modeled among all 3347 KoGES study participants, with the PSQI measured in at least three examinations [26]. We used latent class growth modeling, a group-based modeling approach (SAS Proc Traj), to identify subgroups that share a similar underlying trajectory in sleep quality [27]. The model assumes that the population consists of multiple trajectory groups, rather than simply fitting the overall population mean [28]. We used a censored normal model with PSQI scores, and the time scale was age during the survey examination [27]. A group-based trajectory method is a statistical method for analyzing the evolution of an outcome over age or time [29]. As aging is one of the prominent factors affecting sleep quality [30], we chose age as a time scale for investigating sleep quality patterns: for example, 10-year sleep quality changes in individuals aged 40 to 50 years cannot be treated equally as changes in individuals aged 60 to 70 years. Bayesian information criteria were used to determine the optimal number and shapes of the trajectory groups: a smaller value indicated a better model fit [28]. We calculated the posterior predicted probability for each participant of the five trajectory groups and assigned them to the trajectory group with the greatest posterior probability of membership [31]. The average posterior probabilities of ≥0.70 for each trajectory group indicate high internal reliability within each trajectory and sufficient discrimination of individuals with different sleep quality patterns between trajectories [32].

We used the analysis of variance for normally distributed continuous variables, the Kruskal-Wallis test for skewed distributed variables, and the chi-square test for categorical variables to compare the baseline characteristics of participants in the five sleep quality trajectory groups. We used a multinomial logistic regression model to estimate the associations between baseline SES and trajectory groups of sleep quality. The model evaluated whether SES affects the sleep quality trajectory groups, and odds ratios (OR) with 95% confidence intervals (CIs) were calculated. The associations between baseline SES indices (education and income, and occupation) and sleep quality trajectory groups were analyzed. Potential common confounding factors were chosen based on literature review: biological sex, age, drinking, smoking, moderate exercise, number of family members, and disease diagnosis. All adjusted covariates were obtained from baseline survey data. Additionally, baseline insomnia/depressive symptoms were adjusted considering their time-varying nature [33]. Lastly, educational attainment and occupation were further adjusted when monthly household income was used as the exposure variable and vice versa.

We used mediation models (SAS PROC CAUSALMED) to assess the potential mediation effect of depressive symptoms on the association between baseline SES and longitudinal sleep quality patterns [34]. The outcome variable, sleep quality pattern, was dichotomized (normal-stable vs. moderate-stable, normal-stable vs. poor to moderate, normal-stable vs. developing to poor, and normal-stable vs. severely poor-stable) because of its limitation in the statistical procedure [34]. PROC CAUSALMED estimates causal mediation effects and CIs for the effects based on the maximum likelihood estimates. Alternatively, we utilized 1000 bootstrap resampling to compute CIs for causal mediation effects considering our small sample sizes (e.g. developing to poor trajectory categories) [35]. The factors adjusted in the mediation analyses were the same as those in the main analyses. Lastly, percentages of the total effect that are attributed to mediation and interaction and the percentage of the total effect eliminated by controlling the mediator level were also calculated [34]. In addition, we conducted sensitivity analyses with BDI scores calculated without sleep-related items to confirm the robustness of our findings. We deleted the corresponding scores for the following responses: “I don’t sleep as well as I used to,” “I wake up 1–2 hours earlier than usual and find it hard to get back to sleep,” or “I wake up several hours earlier than I used to and cannot get back to sleep.”

All statistical analyses were performed using SAS software version 9.4 (SAS Institute, Inc., Cary, NC, USA). A two-sided *P*-value of <0.05 was considered significant.

### Ethics

Data in this study were from the KoGES (4851-302), National Research Institute of Health, Centers for Disease Control and Prevention, Ministry for Health and Welfare, Republic of Korea. Our data from the KoGES are freely available if researchers submit appropriate institutional review board clearance to the Korea Centers for Disease Control and Prevention. This study was approved by the Institutional Review Board of Severance Hospital at Yonsei University College of Medicine (Y-2019-0095).

## Results

**Figure 2.**
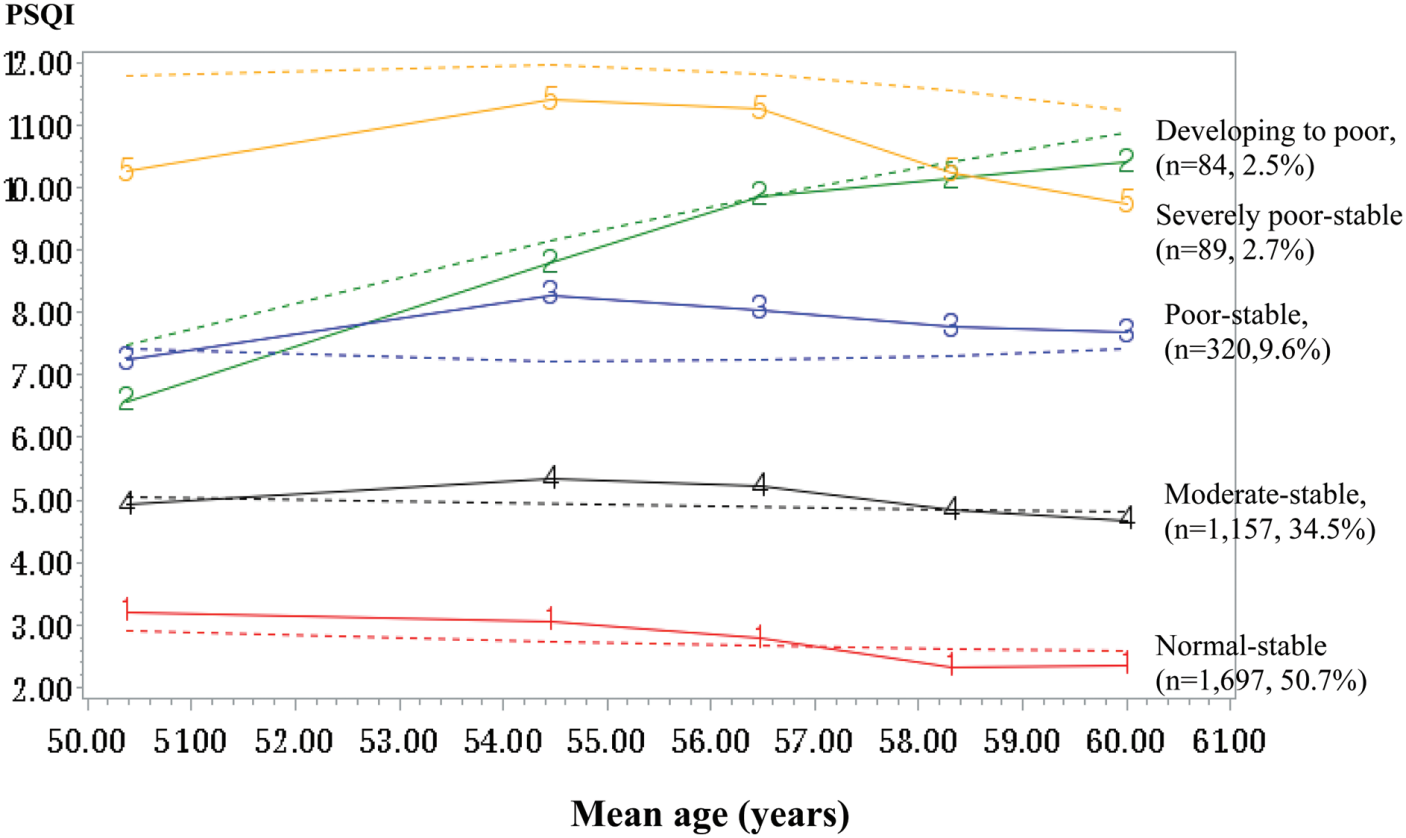
Trajectory groups of sleep quality over 12 years.

We identified five distinct sleep quality trajectories in the middle-aged South Korean participants (Figure 2). Of the 3347 participants, 1697 (normal-stable, 50.7%) had low PSQI scores, 1157 (moderate-stable, 34.5%) had moderate PSQI scores, 320 (poor-stable, 9.6%) had poor PSQI scores, 84 (developing to poor, 2.5%) had increasingly poorer PSQI scores, and 89 still had severely high PSQI scores (severely poor-stable, 2.7%). The average posterior probabilities for all trajectory groups were ≥0.70 (normal-stable: 0.88, moderate-stable: 0.78, poor-stable: 0.77, developing to poor: 0.77, and severely poor-stable: 0.82).

Table 1 presents the descriptive characteristics of study participants stratified according to the sleep quality trajectory. The participants in the poor-stable, developing to poor, and severely poor-stable groups were more likely to be women, older, with lower education, and lower household income than those in the normal-stable group. They were also more likely to be depressed at year 4 and to have shorter sleep durations than those in the normal-stable group. No statistical differences were observed in the disease diagnosis, body mass index, and biologic markers at baseline among the sleep quality groups.

**Table 1.** Baseline characteristic of the participants according to trajectories of sleep quality

	Sleep quality patterns determined by PSQI scores					*P*
	Normal-stable (*n* = 1697)	Moderate-stable (*n* = 1157)	Poor-stable (*n* = 320)	Developing to poor (*n* = 84)	Severely poor- stable (*n* = 89)	
Men, %	984 (58.0)	555 (48.0)	111 (34.7)	21 (25.0)	30 (33.7)	<0.001
Age, years	47.94 ± 7.11	49.09 ± 7.38	50.04 ± 7.77	49.64 ± 7.24	49.44 ± 8.05	<0.001
Education, %						
Lower: high school, or less	1295 (76.4)	891 (77.0)	275 (85.9)	77 (91.7)	79 (89.8)	<0.001
Higher: college, or above	400 (23.6)	266 (23.0)	45 (14.1)	7 (8.3)	9 (10.2)	
Monthly household income, %						
<$2500	1144 (67.8)	799 (69.6)	238 (75.1)	67 (79.8)	71 (79.8)	0.004
≥$2500	544 (32.2)	349 (30.4)	79 (24.9)	17 (20.2)	18 (20.2)	
Occupation, %						
Unemployment (homemaker, etc.)	840 (49.6)	659 (57.1)	227 (71.4)	57 (67.9)	58 (65.2)	<0.001
Manual labor	590 (34.8)	343 (29.7)	64 (20.1)	22 (26.2)	25 (28.1)	
Professional labor	265 (15.6)	152 (13.2)	27 (8.5)	5 (6.0)	6 (6.7)	
Currently married, %	1622 (95.6)	1082 (93.5)	289 (90.3)	81 (96.4)	83 (93.3)	0.011
Currently smoking, %	419 (24.8)	222 (19.1)	65 (20.4)	11 (13.3)	17 (19.1)	<0.001
Currently drinking, %	910 (53.7)	604 (52.2)	148 (46.3)	36 (42.9)	38 (42.7)	0.013
Moderate exercise, %	648 (38.6)	448 (39.4)	134 (42.1)	26 (32.5)	38 (43.2)	0.481
Disease diagnosis*, %	326 (19.2)	253 (21.8)	75 (23.1)	21 (25.0)	20 (22.5)	0.234
Sleep drug intake^†^, %	42 (2.5)	63 (5.5)	44 (13.8)	15 (18.3)	31 (34.8)	<0.001
Sleep duration, hour	6.9 ± 1.12	6.52 ± 1.22	6.14 ± 1.28	6.22 ± 1.63	6.18 ± 1.55	<0.001
BDI^‡^	5 [2-9]	7 [4-12]	9.5 [6-15]	10 [7-16]	14 [7-20]	<0.001
BMI, kg/m^2^	24.67 ± 2.83	24.81 ± 2.89	24.65 ± 2.97	24.48 ± 2.92	24.69 ± 2.69	0.676
Total cholesterol, mg/dL	196.12 ± 35.3	196.69 ± 35.7	195.74 ± 33.56	193.74 ± 32.7	192.78 ± 34.89	0.821
HDL cholesterol, mg/dL	44.36 ± 9.52	44.84 ± 10.01	45.28 ± 9.59	45.61 ± 9.98	46.15 ± 9.48	0.187
LDL cholesterol, mg/dL	121.27 ± 31.43	121.28 ± 30.82	120.21 ± 30.96	117.04 ± 29.15	120.51 ± 32.49	0.777
SBP, mmHg	116.34 ± 16.12	116.63 ± 16.85	116.47 ± 17.73	117.39 ± 17.62	114.01 ± 16.48	0.665
DBP, mmHg	78.27 ± 11.19	77.91 ± 11.05	77.12 ± 11.64	77.86 ± 12.26	76.45 ± 11.65	0.320
Triglyceride, mg/dL	132 [97-186]	134 [97-186]	140 [97-187]	138 [100-189]	121 [89-158]	0.173
HbA1c, %	5.5 [5.3-5.8]	5.6 [5.3-5.8]	5.5 [5.3-5.8]	5.5 [5.3-5.8]	5.5 [5.3-5.9]	0.631
Fasting insulin, uIU/mL	6.8 [5.0-9.1]	6.9 [5.1-9.2]	6.6 [4.7-9.0]	7.1 [5.0-9.6]	6.5 [5.0-8.8]	0.437
Fasting glucose, mg/dL	83 [78-91]	83 [78-92]	82 [77-88]	83.5 [77-90]	82 [76-90]	0.226
High-sensitivity C-reactive protein	0.14 [0.07-0.23]	0.13 [0.06-0.23]	0.14 [0.06-0.25]	0.15 [0.06-0.23]	0.09 [0.04-0.18]	0.099

Abbreviations: BMI, body mass index; HDL, high-density lipoprotein; LDL, low-density lipoprotein; SBP, systolic blood pressure; DBP, diastolic blood pressure; HbA1c, hemoglobin A1c.

*Including cardiovascular disease (coronary artery, myocardial infarction, and cerebrovascular diseases), hypertension, diabetes, dyslipidemia, and cancer.

^†^Including past and current users.

^‡^Assessed at years 4.

Table 2 presents the associations between baseline education/monthly household income and sleep quality trajectory groups. Overall, neither of the SES level indicators were associated with longitudinal sleep quality patterns. In addition, the overall association between occupation and longitudinal sleep quality was not observed (Supplementary Table 2).

**Table 2.** Association between SES and sleep quality patterns

	Trajectory groups of sleep quality											
	Moderate-stable (*n* = 1157) vs. normal-stable (*n* = 1697)			Poor-stable (*n* = 320) vs. normal-stable (*n* = 1697)			Developing to poor (*n* = 84) vs. normal-stable (*n* = 1697)			Severely poor-stable (*n* = 89) vs. Normal-stable (*n* = 1697)		
	*n*	OR	95% CI	*n*	OR	95% CI	*n*	OR	95% CI	*n*	OR	95% CI
Education attainment*												
Lower attainment^‡^ (*n* = 2617)	891	0.82	(0.68 to 1.00)	275	1.04	(0.72 to 1.50)	77	2.09	(0.87 to 5.02)	79	1.45	(0.69 to 3.06)
Higher attainment^§^ (*n* = 727)	266	1.00	Reference	45	1.00	Reference	7	1.00	Reference	9	1.00	Reference
Monthly household income^†^												
Lower income^||^ (*n* = 2319)	799	0.99	(0.83 to 1.18)	238	1.07	(0.79 to 1.45)	67	1.31	(0.73 to 2.35)	71	1.49	(0.84 to 2.64)
Higher income^¶^ (*n* = 1007)	349	1.00	Reference	79	1.00	Reference	17	1.00	Reference	18	1.00	Reference

*Adjustments for sex, age, job, monthly household income, drinking, smoking, moderate exercise, number of family members, disease diagnosis, insomnia symptom, and depressive mood at baseline.

^†^Adjustments for sex, age, job, education attainment, drinking, smoking, moderate exercise, number of family members, disease diagnosis, insomnia symptom, and depressive mood at baseline.

^‡^High school or less.

^§^College or above.

^||^<$2500.

^¶^≥$2500.

The mediation effects of depressive symptoms between SES (education/monthly household income) and longitudinal sleep quality patterns are presented in Table 3. There were no direct or total effects of educational attainment on longitudinal sleep quality. However, there were indirect effects of lower educational attainment on longitudinal sleep quality through depressive symptoms (moderate-stable: OR = 1.05, 95% CI = 1.01 to 1.09; poor-stable: OR = 1.10, 95% CI = 1.04 to 1.20; developing to poor: OR = 1.12, 95% CI = 1.04 to 1.24; and severely poor-stable: OR = 1.17, 95% CI = 1.06 to 1.32). Similarly, lower household income was associated with worse sleep quality, only because of depressive symptoms (moderate-stable: OR = 1.05, 95% CI = 1.02 to 1.09; poor-stable: OR = 1.09, 95% CI = 1.03 to 1.16; developing to poor: OR = 1.09, 95% CI = 1.03 to 1.18; and severely poor-stable: OR = 1.15, 95% CI = 1.07 to 1.29). However, no significant mediating role of depressive symptoms was found for the associations between occupation and longitudinal sleep quality (Supplementary Table 3). Percentage mediated, percentage due to interaction, and percentage eliminated are provided in Supplementary Table 4. If the mediated effect has a different sign than other direct effect in a model, the absolute values of the direct and indirect effects should be considered prior to calculating the proportion mediated [35] (e.g. lower income; OR for direct effect of SES: 0.93 and OR for indirect effect of SES through depressive symptom: 1.05). Sensitivity analyses with BDI scores eliminating the sleep-related items were similar to the main results (Supplementary Table 5).

**Table 3.** Association between SES and sleep quality patterns mediated by depressive symptom at years 4

	Trajectory groups of sleep quality							
	Moderate-stable (*n* = 1157) vs. normal-stable (*n* = 1697)		Poor-stable (*n* = 320) vs. normal-stable (*n* = 1697)		Developing to poor (*n* = 84) vs. normal-stable (*n* = 1697)		Severely poor-stable (*n* = 89) vs. normal-stable (*n* = 1697)	
	OR	95% CI	OR	95% CI	OR	95% CI	OR	95% CI
Education attainment*								
Lower attainment^‡^ (*n* = 2617) vs. higher attainment^§^ (ref, *n* = 727)								
Total effect	0.80	(0.64 to 1.01)	1.15	(0.72 to 1.83)	1.55	(0.64 to 6.03)	2.69	(1.14 to 9.43)
Natural direct effect	0.76	(0.62 to 0.96)	1.04	(0.66 to 1.64)	1.38	(0.58 to 5.09)	2.30	(0.97 to 8.01)
Natural indirect effect	1.05	(1.01 to 1.09)	1.10	(1.04 to 1.20)	1.12	(1.04 to 1.24)	1.17	(1.06 to 1.32)
Monthly household income^†^								
Lower income^||^ (*n* = 2319) vs. Higher income^¶^ (ref, *n* = 1007)								
Total effect	0.98	(0.81 to 1.19)	0.998	(0.72 to 1.41)	0.97	(0.46 to 2.07)	1.20	(0.58 to 2.92)
Natural direct effect	0.93	(0.78 to 1.14)	0.91	(0.67 to 1.29)	0.90	(0.42 to 1.91)	1.04	(0.49 to 2.49)
Natural indirect effect	1.05	(1.02 to 1.09)	1.09	(1.03 to 1.16)	1.09	(1.03 to 1.18)	1.15	(1.07 to 1.29)

In total, 395 data were deleted due to no measurements of BDI scores at years 4.

*Adjustments for sex, age, job, monthly household income, drinking, smoking, moderate exercise, disease diagnosis, insomnia symptom, and depressive mood at baseline.

^†^Adjustments for sex, age, job, education attainment, drinking, smoking, moderate exercise, number of family members, disease diagnosis, insomnia symptom, and depressive mood at baseline.

^‡^High school or less.

^§^College or above.

^||^<$2500.

^¶^≥$2500.

## Discussion

In this study, we identified five distinct trajectories of sleep quality over a 12-year period in a middle-aged Korean adult population: normal-stable, moderate-stable, poor to moderate, developing to poor, and severely poor-stable patterns. Although there were no significant overall or direct associations between SES levels and longitudinal sleep quality patterns, depressive symptoms fully mediated the association between SES (education attainment and monthly household income) and longitudinal sleep quality patterns.

The prevalence of insomnia symptoms in the present study was similar to that reported in a previous South Korean population study [6]. In the current study, 15% of the participants experienced deleterious changes in their sleep quality or maintained poor sleep quality. Consistent with our results, 20% of 5000 South Korean individuals aged 20–69 years, who were included in a study using a stratified, multistage random sampling method based on the geographical region, residence, sex, age, occupation, and income, reported insomnia symptoms (i.e. difficulty in waking up or getting back to sleep at night) [6]. Changes in sleep quality (developing to poor) among people with insomnia symptoms were also in line with the findings of a previous study [16]. Among 250 Canadian individuals, changes in sleep quality in people with insomnia were investigated during a 3-year period; one-half of the sample with insomnia at baseline experienced good sleep quality at least once, while some of them eventually developed insomnia during subsequent assessments [16]. In addition, participants in the developing to poor and severely poor-stable groups in this study were more likely to be women and older than those in other sleep pattern groups. In support thereof, research has shown that aging is a prominent factor that affects sleep quality and that women have a higher prevalence of insomnia than men in general [30].

In current analyses, the overall association between SES level and longitudinal sleep quality patterns over 12 years was not apparent, which did not align with previous studies [8, 9]. In a cross-sectional study of 301 women aged ≥55 years, higher education attainment was associated with reduced sleep latency based on PSQI scores [8]. Additionally, researchers have shown that highly educated people have higher chances of access to obtaining greater knowledge about sleep hygiene practices and strategies that can improve their sleep environment as well as better recognition of the importance of sleep for health [10, 36]. Therefore, highly educated people may proactively seek help for their sleep problems [36]. In addition, among 160 000 participants aged ≥18 years from 36 states/regions across the United States, people with lower SES frequently reported sleep complaints, such as trouble falling asleep or staying asleep, in a cross-sectional manner [9]. Notwithstanding, these previous studies only presented the cross-sectional associations between the level of SES and sleep quality [8–10], whereas we investigated the further impact of SES levels on sleep quality changes.

In this study, depressive symptoms significantly mediated the association between SES level (education/household income) and longitudinal sleep quality. These findings suggest that low SES can decrease sleep quality over time through depressive symptoms, even in a population wherein a link between SES and sleep quality is not clearly observable. Several previous studies have reported that people with lower education or income are more likely to be depressed [11] and that lower education or income may affect sleep quality [37]. According to a community-based longitudinal study of 7000 populations from California, SES measured by education or income presented dose-response relationships with prevalent and incident depression status [38]. This could be due to economic stress or disadvantages across the life course that people with lower SES may experience [39]. Approximately 14%–21% of people with insomnia were reported to experience major depression in two community-based epidemiological studies, compared with those without sleep complaints [13]. This could be supported by the findings of our study in that participants with worse sleep quality patterns had higher BDI scores at year 4, which indicate more severe depressive symptoms. Based on the cross-sectional understanding of SES or mood status effects on sleep quality, we further reported that the impact of lower SES levels and depressive symptoms related to longitudinal poor sleep quality.

### Strengths and limitations

The strengths of this study are the prospective nature of the study and the repeated assessments of validated sleep quality data over 12 years. Furthermore, we investigated the longitudinal changes of sleep quality; since previous studies utilized limited assessments of sleep quality [3, 14] and limited observation time [16], it was difficult to report longitudinal sleep quality changes. In addition, we presented the mediation effect of depressive symptoms in the association between baseline SES and longitudinal sleep quality patterns. However, several limitations need to be considered. First, the observational study design may limit causal inference of the association between SES and sleep quality patterns mediated by depressive symptoms. However, since we assumed that depressive symptoms at year 4 would be a potential mediator, the temporality of the association may give benefits to the causality of the association [40]. Second, we could not find a significant association among occupation, depressive symptoms, and longitudinal sleep quality. However, the cultural context of Korea may explain the null results for occupation: women are primarily in charge of housework in Korea, and this may lead to differing distributions in occupation [41]. This could be supported by our data in that almost 68% of women were included as “homemakers,” whereas around 67% of men were categorized as manual labor or professional labor (Supplementary Table 1). However, we could not conduct analyses stratified by sex due to small sample sizes. Therefore, utilizing an occupational classification as a socioeconomic indicator may not adequately capture disparities in working conditions across sex [42]. Furthermore, people who are not currently employed (i.e. homemakers) are not easily assigned to occupation classification [42]; for example, homemakers and retired persons cannot be simply placed together with the “unemployed.” Considering the sex-specific nature of occupation, we may not have completely reflected all aspects thereof as a proxy for SES level in our data. Third, the study results should be interpreted with caution with regard to generalizability. The participants of our study are representative of Ansan city, an industrialized community in South Korea; therefore, it would be difficult to generalize the study results to all middle-aged Korean adults. The study participants tended to be of higher SES and have healthier lifestyle habits compared with the national representative sample for Korea [43] (Supplementary Table 6). Fourth, a residual confounding may still exist, even though we have adjusted sex considering its different proportion in each sleep pattern. There might be a chance to conceal the influence of lifestyle behavior, which is highly correlated to sex (i.e. current smoking or alcohol drinking) on sleep and mood, and that may partially affect the generalizability of the findings. Fifth, although we used longitudinal data within the 12-year period, sleep quality patterns were derived using a limited number of sleep quality measurements. However, sleep quality changes, especially those in people with insomnia [16], were reflected in our findings on sleep quality patterns. In addition, trajectory groups in our study showed posterior probabilities ≥0.7, which indicates sufficient discrimination of the participants in each trajectory group. Finally, not all participants had available PSQI information at all examination periods. However, missing PSQI data (i.e. three or four measurements) are unlikely to have altered our findings, as the mean number of PSQI measurements was 4.3 to 4.6 according to sleep quality trajectory groups.

## Conclusions

Sleep quality is highly related to health in both physical and mental aspects. Despite its importance, only a few existing studies have reported on the longitudinal changes in sleep quality. During a 12-year span, we identified five heterogeneous sleep quality trajectories among 3347 middle-aged Korean individuals. Baseline SES levels were indirectly associated with longitudinal sleep quality patterns; a substantial amount of this association was mediated by depressive symptoms. Lower education attainment and household income have an impact on psychiatric status, which in turn leads to an increase in worse sleep quality (e.g. developing to poor or severely poor-stable levels). Overall, our findings provide further information on the natural history of sleep quality and psychiatric status according to SES. Furthermore, our discovery of a mediation effect of depressive symptoms between SES level and sleep quality holds important clinical significance: the mental health of people with low SES matters to their physical health. Our findings may provide evidence of mental health vulnerability in people with lower SES and its detrimental effects on longitudinal sleep quality, which is an important factor for overall health. Therefore, at the community level, mental health care should be reconsidered in people with low SES in primary care.

## Authors Contributions

The authors of this study have involved in the following activities: G.B.L.: conceptualization, data curation, writing—original draft, and writing—review and editing; Y.J.J.: data curation and writing—review and editing; H.C.K.: writing—review and editing; and S.J.J.: supervision, conceptualization, data curation, writing—original draft, and writing—review and editing.

## Funding

This research was supported by Basic Science Research Program through the National Research Foundation of Korea (NRF) funded by the Ministry of Science and Information and Communication Technology (2020R1C1C1003502) awarded to S.J.J.

*Conflict of interest statement*. Authors declare no potential conflicts of interest.

## Supplementary Material

zsab044_suppl_Supplementary_Materials
