# Supplementary material for: Association between socioeconomic status and longitudinal sleep quality patterns mediated by depressive symptoms
Source: Sleep. 2021 Feb 25;44(8):zsab044. doi: 10.1093/sleep/zsab044 (PMC8361348; doi:10.1093/sleep/zsab044)
Supplement: zsab044_suppl_Supplementary_Materials [file zsab044_suppl_supplementary_materials.docx]

| Supplementary table 1. Distribution of occupations by sex in KoGES-Ansan data | | | | | |
| --- | --- | --- | --- | --- | --- |
| Occupation Classification | Sex | | | | |
|  | Men | |  | Women | |
| Homemaker | 2 | (0.12) |  | 1,111 | (67.62) |
| Office worker | 226 | (13.32) |  | 27 | (1.64) |
| Farmworker | 11 | (0.65) |  | 7 | (0.43) |
| Self-employed | 562 | (33.12) |  | 185 | (11.26) |
| Sales | 26 | (1.53) |  | 46 | (2.80) |
| Factory worker | 154 | (9.07) |  | 53 | (3.23) |
| Expertise | 165 | (9.72) |  | 37 | (2.25) |
| Etc. | 551 | (32.47) |  | 177 | (10.77) |
| Total | 1,697 | 100 |  | 1,643 | 100 |
| Notes. homemaker and etc. were classified as “unemployed”; office worker and expertise were classified as “professional laborers”; others were classified as “manual laborers.” | | | | | |
|  |  |  |  |  |  |
|  |  |  |  |  |  |
|  |  |  |  |  |  |

| Supplementary table 2. Association between occupation and sleep quality patterns | | | | | | | | | | | | | | | | | | | | | | | | |
| --- | --- | --- | --- | --- | --- | --- | --- | --- | --- | --- | --- | --- | --- | --- | --- | --- | --- | --- | --- | --- | --- | --- | --- | --- |
|  | Trajectory Groups of Sleep Quality | | | | | | | | | | | | | | | | | | | | | | | |
|  | Moderate-stable (n=1,157) vs. Normal-stable (n=1,697) | | | | |  | Poor-stable (n=320) vs. Normal-stable (n=1,697) | | | | |  | Developing to Poor (n=84) vs. Normal-stable (n=1,697) | | | | |  | Severely poor-stable (n=89) vs. Normal-stable (n=1,697) | | | | | |
|  | n | OR | 95% CI | | |  | n | OR | 95% CI | | |  | n | OR | 95% CI | | |  | n | OR | 95% CI | | |  |
| Occupation |  |  |  |  |  |  |  |  |  |  |  |  |  |  |  |  |  |  |  |  |  |  |  |  |
| Unemployed (n=1,841) | 659 | 1.00 | reference | | |  | 227 | 1.00 | reference | | |  | 57 | 1.00 | reference | | |  | 58 | 1.00 | reference | | |  |
| Manual Labor (n=1,044) | 343 | 0.91 | (0.75 | **-** | 1.11) |  | 64 | 0.59 | (0.42 | **-** | 0.82) |  | 22 | 0.94 | (0.53 | **-** | 1.68) |  | 25 | 0.97 | (0.56 | **-** | 1.67) |  |
| Professional Labor (n=455) | 152 | 0.94 | (0.72 | **-** | 1.22) |  | 27 | 0.66 | (0.41 | **-** | 1.09) |  | 5 | 0.84 | (0.31 | **-** | 2.33) |  | 6 | 0.74 | (0.29 | **-** | 1.91) |  |
| Adjustments for sex, age, education attainment, monthly household income, education attainment, drinking, smoking, moderate exercise, number of family members, disease diagnosis, insomnia symptom, and depressive mood at baseline | | | | | | | | | | | | | | | | | | | | | | | | |

| Supplementary table 3. Association between occupation and sleep quality patterns mediated by depressive symptoms at year 4 | | | | | | | | | | | | | | | | | | | | | |
| --- | --- | --- | --- | --- | --- | --- | --- | --- | --- | --- | --- | --- | --- | --- | --- | --- | --- | --- | --- | --- | --- |
|  | Trajectory Groups of Sleep Quality | | | | | | | | | | | | | | | | | | | | |
|  | Moderate-stable (n=1,157) vs. Normal-stable (n=1,697) | | | |  | Poor-stable (n=320) vs. Normal-stable (n=1,697) | | | |  | Developing to Poor (n=84) vs. Normal-stable (n=1,697) | | | |  | Severely poor-stable (n=89) vs. Normal-stable (n=1,697) | | | | |  |
|  | OR | 95% CI | | |  | OR | 95% CI | | |  | OR | 95% CI | | |  | OR | 95% CI | | | |  |
| Occupation |  |  |  |  |  |  |  |  |  |  |  |  |  |  |  |  |  |  |  |  |  |
| Manual labor (n=1,044) vs. unemployed (ref, n=1,841) |  |  |  |  |  |  |  |  |  |  |  |  |  |  |  |  |  |  |  |  |  |
| Total effect | 0.91 | (0.71 | **-** | 1.10) |  | 0.56 | (0.34 | **-** | 0.78) |  | 1.02 | (0.33 | **-** | 1.71) |  | 0.63 | (0.14 | **-** | 1.12) |  |  |
| Natural direct effect | 0.87 | (0.69 | **-** | 1.06) |  | 0.56 | (0.34 | **-** | 0.78) |  | 1.01 | (0.33 | **-** | 1.68) |  | 0.60 | (0.14 | **-** | 1.06) |  |  |
| Natural indirect effect | 1.04 | (0.998 | **-** | 1.07) |  | 0.99 | (0.92 | **-** | 1.06) |  | 1.02 | (0.96 | **-** | 1.08) |  | 1.05 | (0.94 | **-** | 1.15) |  |  |
| Professional labor (n=455) vs. unemployed (ref, n=1,841) |  |  |  |  |  |  |  |  |  |  |  |  |  |  |  |  |  |  |  |  |  |
| Total effect | 1.01 | (0.70 | **-** | 1.31) |  | 0.52 | (0.20 | **-** | 0.84) |  | 0.67 | (0.00 | **-** | 1.60) |  | 0.59 | (0.00 | **-** | 1.39) |  |  |
| Natural direct effect | 1.00 | (0.71 | **-** | 1.30) |  | 0.54 | (0.21 | **-** | 0.87) |  | 0.69 | (0.00 | **-** | 1.63) |  | 0.60 | (0.00 | **-** | 1.40) |  |  |
| Natural indirect effect | 1.00 | (0.96 | **-** | 1.05) |  | 0.96 | (0.87 | **-** | 1.06) |  | 0.98 | (0.88 | **-** | 1.08) |  | 0.99 | (0.86 | **-** | 1.11) |  |  |
| Notes: 395 data were deleted due to no measurements of BDI scores at year 4. Adjustments for sex, age, job, monthly household income, education attainment, drinking, smoking, moderate exercise, number of family members, disease diagnosis, insomnia symptom, and depressive mood at baseline | | | | | | | | | | | | | | | | | | | | | |

| Supplementary table 4. Association between Socioeconomic Status and Sleep Quality Patterns Mediated by Depressive Symptom at Years 4 | | | | | | | | | | | | | | | | | | | |
| --- | --- | --- | --- | --- | --- | --- | --- | --- | --- | --- | --- | --- | --- | --- | --- | --- | --- | --- | --- |
|  | Trajectory Groups of Sleep Quality | | | | | | | | | | | | | | | | | | |
|  | Moderate-stable (n=1,157) vs Normal-stable (n=1,697) | | | |  | Poor-stable (n=320) vs Normal-stable (n=1,697) | | | |  | Developing to Poor (n=84) vs Normal-stable (n=1,697) | | | |  | Severely poor-stable (n=89) vs Normal-stable (n=1,697) | | | |
|  | OR | 95% CI | | |  | OR | 95% CI | | |  | OR | 95% CI | | |  | OR | 95% CI | | |
| Education attainment* |  |  |  |  |  |  |  |  |  |  |  |  |  |  |  |  |  |  |  |
| Lower attainment^a^ (n=2,617) vs Higher attainment^b^ (ref, n=727) |  |  |  |  |  |  |  |  |  |  |  |  |  |  |  |  |  |  |  |
| Total effect | 0.80 | (0.64 | **-** | 1.01) |  | 1.15 | (0.72 | **-** | 1.83) |  | 1.55 | (0.64 | **-** | 6.03) |  | 2.69 | (1.14 | **-** | 9.43) |
| Natural direct effect | 0.76 | (0.62 | **-** | 0.96) |  | 1.04 | (0.66 | **-** | 1.64) |  | 1.38 | (0.58 | **-** | 5.09) |  | 2.30 | (0.97 | **-** | 8.01) |
| Natural indirect effect | 1.05 | (1.01 | **-** | 1.09) |  | 1.10 | (1.04 | **-** | 1.20) |  | 1.12 | (1.04 | **-** | 1.24) |  | 1.17 | (1.06 | **-** | 1.32) |
| Percentage mediated | - | | | |  | 72.4 | (-806.5 | **-** | 423.5) |  | 29.8 | (-237.5 | **-** | 210.9) |  | 22.8 | (7.0 | **-** | 82.0) |
| Percentage due to interaction | 8.3 | (2.5 | **-** | 21.5) |  | 5.0 | (-58.6 | **-** | 192.4) |  | 10.6 | (-16.2 | **-** | 51.5) |  | 23.9 | (5.7 | **-** | 42.4) |
| Percentage eliminated | - | | | |  | 74.6 | (-704.8 | **-** | 403.3) |  | 32.1 | (-201.9 | **-** | 210.4) |  | 33.8 | (19.0 | **-** | 84.6) |
| Monthly household income† |  |  |  |  |  |  |  |  |  |  |  |  |  |  |  |  |  |  |  |
| Lower income^c^ (n=2,319) vs Higher income^d^ (ref, n=1,007) |  |  |  |  |  |  |  |  |  |  |  |  |  |  |  |  |  |  |  |
| Total effect | 0.98 | (0.81 | **-** | 1.19) |  | 0.998 | (0.72 | **-** | 1.41) |  | 0.97 | (0.46 | **-** | 2.07) |  | 1.20 | (0.58 | **-** | 2.92) |
| Natural direct effect | 0.93 | (0.78 | **-** | 1.14) |  | 0.91 | (0.67 | **-** | 1.29) |  | 0.90 | (0.42 | **-** | 1.91) |  | 1.04 | (0.49 | **-** | 2.49) |
| Natural indirect effect | 1.05 | (1.02 | **-** | 1.09) |  | 1.09 | (1.03 | **-** | 1.16) |  | 1.09 | (1.03 | **-** | 1.18) |  | 1.15 | (1.07 | **-** | 1.29) |
| Percentage mediated | - | | | |  | - | | | |  | - | | | |  | 78.0 | (-655.7 | **-** | 438.9) |
| Percentage due to interaction | 25.9 | (47.2 | **-** | 60.3) |  | 863.8 | (-117.8 | **-** | 194.1) |  | 60.8 | (-34.3 | **-** | 131.4) |  | 6.9 | (131.4 | **-** | 222.0) |
| Percentage eliminated | - | | | |  | - | | | |  | - | | | |  | 81.6 | (-546.1 | **-** | 384.2) |
| Notes: 395 data was deleted due to no measurements of BDI scores at years 4 a. High school or less b. College or above c. < $2,500 d. ≥ $2,500 *Adjustments for sex, age, job, monthly household income, drinking, smoking, moderate exercise, disease diagnosis, insomnia symptom, and depressive mood at baseline †Adjustments for sex, age, job, education attainment, drinking, smoking,, moderate exercise, number of family members, disease diagnosis, insomnia symptom, and depressive mood at baseline | | | | | | | | | | | | | | | | | | | |

| Supplementary table 5. Association between socioeconomic status and sleep quality patterns mediated by depressive symptoms at year 4 | | | | | | | | | | | | | | | | | | | | |
| --- | --- | --- | --- | --- | --- | --- | --- | --- | --- | --- | --- | --- | --- | --- | --- | --- | --- | --- | --- | --- |
|  | Trajectory Groups of Sleep Quality | | | | | | | | | | | | | | | | | | | |
|  | Moderate-stable (n=1,157) vs. Normal-stable (n=1,697) | | | |  | Poor-stable (n=320) vs. Normal-stable (n=1,697) | | | |  | Developing to Poor (n=84) vs. Normal-stable (n=1,697) | | | |  | Severely poor-stable (n=89) vs. Normal-stable (n=1,697) | | | |  |
|  | OR | 95% CI | | |  | OR | 95% CI | | |  | OR | 95% CI | | |  | OR | 95% CI | | |  |
| Education attainment* |  |  |  |  |  |  |  |  |  |  |  |  |  |  |  |  |  |  |  |  |
| Lower attainment^a^ (n=2,617) vs. higher attainment^b^ (ref, n=727) |  |  |  |  |  |  |  |  |  |  |  |  |  |  |  |  |  |  |  |  |
| Total effect | 0.80 | (0.63 | **-** | 0.97) |  | 1.15 | (0.66 | **-** | 1.64) |  | 1.58 | (0.09 | **-** | 3.07) |  | 2.61 | (0.00 | **-** | 5.30) |  |
| Natural direct effect | 0.77 | (0.60 | **-** | 0.93) |  | 1.06 | (0.61 | **-** | 1.50) |  | 1.44 | (0.08 | **-** | 2.80) |  | 2.27 | (0.00 | **-** | 4.61) |  |
| Natural indirect effect | 1.04 | (1.01 | **-** | 1.08) |  | 1.09 | (1.02 | **-** | 1.16) |  | 1.10 | (1.02 | **-** | 1.18) |  | 1.15 | (1.04 | **-** | 1.26) |  |
| Monthly household income† |  |  |  |  |  |  |  |  |  |  |  |  |  |  |  |  |  |  |  |  |
| Lower income^c^ (n=2,319) vs. higher income^d^ (ref, n=1,007) |  |  |  |  |  |  |  |  |  |  |  |  |  |  |  |  |  |  |  |  |
| Total effect | 0.98 | (0.79 | **-** | 1.17) |  | 1.01 | (0.65 | **-** | 1.36) |  | 0.98 | (0.33 | **-** | 1.64) |  | 1.25 | (0.31 | **-** | 2.18) |  |
| Natural direct effect | 0.93 | (0.76 | **-** | 1.11) |  | 0.93 | (0.60 | **-** | 1.25) |  | 0.91 | (0.30 | **-** | 1.52) |  | 1.09 | (0.27 | **-** | 1.92) |  |
| Natural indirect effect | 1.05 | (1.02 | **-** | 1.08) |  | 1.09 | (1.02 | **-** | 1.15) |  | 1.08 | (1.01 | **-** | 1.15) |  | 1.14 | (1.04 | **-** | 1.24) |  |
| Notes. 395 data were deleted due to no measurements of BDI scores at year 4 a. High school or less b. College or above c. < $2,500 d. ≥ $2,500 *Adjustments for sex, age, job, monthly household income, drinking, smoking, moderate exercise, disease diagnosis, insomnia symptom, and depressive mood at baseline †Adjustments for sex, age, job, education attainment, drinking, smoking,, moderate exercise, number of family members, disease diagnosis, insomnia symptom, and depressive mood at baseline ‡Sleep-related questionnaire was eliminated from scoring of Beck's Depression Inventory | | | | | | | | | | | | | | | | | | | | |

| Supplementary table 6. Comparison of characteristics of the participants between KoGES-Ansan baseline data and Korea National Health and Nutrition Examination Survey (KNHANES) 2001 data | | | | | | |
| --- | --- | --- | --- | --- | --- | --- |
|  |  |  |  |  |  |  |
|  | Data, aged 40 to 69 years | | | | | |
|  | Ansan KoGES 2001-2002, (n=3,347) | | | Korea National Health and Nutrition Examination Survey (KNHANES) 2001  (n=13,003) | | |
| Men, % | 1,701 |  | (50.8) | 5,701 |  | (49.0) |
| Age, years | 48.62 | ± | 7.33 | 51.72 | ± | 8.64 |
| 40 to < 50 | 2,174 |  | (65.0) | 6,243 |  | (48.0) |
| 50 to < 60 | 800 |  | (23.9) | 3,728 |  | (28.7) |
| 60 to < 70 | 373 |  | (11.1) | 3,032 |  | (23.3) |
| BMI, kg/m² | 24.72 | ± | 2.86 | 24.17 | ± | 3.05 |
| Education, % |  |  |  |  |  |  |
| High school or less | 2,617 |  | (78.3) | 10,913 |  | (84.1) |
| College or above | 727 |  | (21.7) | 2,056 |  | (15.9) |
| Monthly household income, % |  |  |  |  |  |  |
| < $2,500 | 2,319 |  | (69.7) | 10,019 |  | (83.0) |
| ≥ $2,500 | 1,007 |  | (30.3) | 2060 |  | (17.1) |
| Occupation, % |  |  |  |  |  |  |
| Unemployment (homemaker or etc.) | 1,841 |  | (55.1) | 4,673 |  | (36.1) |
| Manual labor | 1,044 |  | (31.3) | 6,773 |  | (52.3) |
| Professional labor | 455 |  | (13.6) | 1,500 |  | (11.6) |
| Currently married, % | 3,130 |  | (93.5) | 11,191 |  | (86.1) |
| Currently smoking, % | 734 |  | (22.0) | 1,160 |  | (30.0) |
| Currently drinking, % | 1,736 |  | (51.9) | 2,263 |  | (65.2) |
| Disease diagnosis*, % | 602 |  | (18.0) | 1,799 |  | (15.5) |
| Sleep duration, hour | 6.66 | ± | 1.23 | 7.21 | ± | 6.75 |
| SBP, mmHg | 116.42 | ± | 16.58 | 126.23 | ± | 19.61 |
| DBP, mmHg | 77.98 | ± | 11.22 | 79.93 | ± | 11.52 |
| LDL cholesterol, mg/dL | 119.2 |  | [99.2-140.8] | 119 |  | [97.4-140.4] |
| Triglyceride, mg/dL | 133 |  | [97-186] | 130 |  | [92-191] |
| HbA1c, % | 5.5 |  | [5.3-5.8] | 6 |  | [5.0-6.0] |
| Fasting glucose, mg/dL | 83 |  | [78-91] | 96 |  | [88-107] |
| Abbreviations: BMI, body mass index; SBP, systolic blood pressure; DBP, diastolic blood pressure; LDL, low-density lipoprotein; HbA1c, hemoglobin A1c.  *including coronary artery, myocardial infarction, hypertension, diabetes, and cancer. | | | | | | |
|  |  |  |  |  |  |  |
|  |  |  |  |  |  |  |
|  |  |  |  |  |  |  |
